# U.S. politics as a reminder of historical losses in American Indian Adults: Implications for political participation

**DOI:** 10.1371/journal.pone.0336671

**Published:** 2025-12-08

**Authors:** Zachary J. Wood, Peter J. Helm, Neha A. John-Henderson

**Affiliations:** Department of Psychology, Montana State University, Bozeman, Montana; Universidade Federal do Tocantins, BRAZIL

## Abstract

Historical loss (HL) is a contributor to experiences of historical trauma and its negative impacts on American Indians. The relationship between HL, U.S. politics and political participation among American Indians has not been examined. Minority groups can become politically active through perceiving discrimination or injustices against them. Measures of HL contain items related to the discrimination and injustices that American Indians have experienced. While the Historical Loss Scale (HLS) measures the frequency with which American Indians think about HL, it does not measure the degree to which U.S. Politics may act as a reminder of HL. Here, we introduce an adaptation of the HLS: the U.S. Politics as a Loss Reminder Scale (USPLRS), measuring the degree to which U.S. politics may remind American Indian adults of HL, with higher scores reflecting a greater association between U.S. politics and HL. A sample of 877 American Indian adults completed a series of questionnaires including the HLS, USPLRS, and measures of political participation during the November 2020 national election cycle. Results revealed a 3-factor structure of the USPLRS related to losses due to Government Mistreatment, Interpersonal Loss, and Respect. Scores on both the HLS and USPLRS were positively associated with political participation, with the USPLRS exhibiting incremental validity beyond the HLS.

## Introduction

The experiences of American Indians are shaped by the long-lasting effects of genocidal governmental policies, including colonization, violence, and genocide, which have resulted in the loss of land, language, and culture [1]. Historical trauma is defined as “cumulative emotional and psychological wounding over the lifespan and across generations, emanating from massive group trauma experiences” [2]. The degree to which American Indians think about the loss of land, life, culture, and other aspects related to the past and present atrocities committed against their people is posited to be one contributor to current experiences of historical trauma and has been coined historical loss (HL) [3].

To measure HL, Whitbeck et al. [1] developed and validated the Historical Loss Scale (HLS). The HLS assesses the frequency of thoughts related to losses resulting from European colonization and consists of 12 items measured on a 6-point scale (1-several times a day to 6-never). Most of the research utilizing the HLS has examined its relationship with health outcomes. Research has shown that thoughts about historical losses can be distressing for American Indians, and thus, historical loss can be conceptualized as a stressor [4]. Overall, scores on the HLS have been associated with negative mental health outcomes such as increased anxiety, depression, thoughts of suicide, substance abuse, and symptoms of post-traumatic stress disorder (PTSD) [5–7]. Scores on the HLS have also been associated with negative physical health outcomes such as increased risk for cardiovascular disease evidenced by increased systolic, and diastolic ambulatory blood pressure for American Indian adults that reported a higher frequency of thoughts about historical losses [8]. However, there remains a significant gap in the literature. Specifically, the relationship between thoughts about historical loss and political participation has not been examined.

Given the historical losses that have occurred due to U.S. governmental policies, the degree to which U.S. politics act as a reminder of these losses may affect political participation among American Indians. For example, American Indians were not granted citizenship until 1924 which coincided with the boarding school era, which aimed to destroy American Indian culture and identities [9]. Despite obtaining citizenship, American Indians were not granted a federally protected right to vote until the passage of the Voting Rights Act of 1965 [10]. However, like other minority groups such as African Americans, in the context of political participation, American Indians faced literacy tests, voter intimidation, and poll taxes [9]. These injustices have shaped relationships with U.S. governmental institutions and may relate to political behavior in multiple ways. The goal of the current paper is not to explain overall participation levels, but to examine a politics-specific reminder of historical loss.

### Importance and implications of political participation among American Indians

Political participation is a fundamental aspect of democracy that enables citizens to influence collective descisions and act collectively to promote change and maintain community values [11,12]. Research on political participation among American Indians suggest that they participate at relatively lower levels than other groups [10,13]. All segments of the population should have the same opportunity to engage in politics to maintain a truly democratic society [14]. For American Indians, ample participation is also necessary to maintain sovereignty and the rights associated with it [15]. Further, American Indians face significant health disparities stemming from historical losses [3,7,6], and thus identifying potential pathways to resolve these disparities is necessary. When controlling for factors such as rates of poverty, smoking, and other health-related factors, recent research has found that lower levels of political participation are associated with an increase in health disparities [16]. Further, political participation has been associated with positive health outcomes such as improvements in community conditions that promote health such as safe streets, updates to housing conditions, and improved sanitation systems [17], and voting has been positively associated with well-being [18]. Understanding political participation among American Indians is important, however, the current study focuses on validating a politics-specific loss-reminder measure (USPLRS) and testing its association with political participation.

### From historical loss to political participation

The aim of the current study is to validate a politics-specific loss reminder measure (USPLRS) and test its association with political participation, distinguishing it from the general frequency of thoughts related to historical loss captured by the HLS. Previous research shows that perceptions of injustices can mobilize political engagement via politicized identity and efficacy processes [19–24]. For American Indians, this is particularly relevant given the historical injustices and discrimination that have occurred stemming from colonization and perpetuated by the U.S. governmental policies [1,2]. This unique history emphasizes the importance of understanding how experiences of injustices and discrimination at the hands of the U.S. government relate to political participation in American Indians.

### Operationalizing political participation

Political participation can include a variety of activities such as voting, discussing politics, participating in political organizations, posting about political issues online, engaging with political news, campaigning, and many other activities [11,25–27]. In the current study, political participation is operationalized as documenting political issues online, participating in political clubs or organizations, reading political news articles, watching political news shows, discussing political issues with friends, and discussing political issues with family members. These activities provide a broad measure of both relatively active and accessible forms of political participation. Importantly, our measures of political participation capture non-electoral activities, rather than electoral turnout (e.g., voting). Thus, we use “political participation” broadly and do not draw inferences about voting behavior [25]; Cortes et al., 2007; [27]).

Prior research suggests two potential pathways linking historical loss and political behavior. First, frequency of thoughts related to historical loss has been associated with distress (e.g., anxiety, depression, PTSD), which may reduce participation [5,7,28]. However, when losses are appraised in explicitly political terms, they can mobilize participation through politicized identity and efficacy processes [19,21–23,29–31]. The current study focused on the second pathway by testing whether politics-specific loss reminders (USPLRS) are associated with greater levels of political participation, while clearly distinguishing them from the general historical loss frequency captured by the HLS [1,4].

### Why a U.S. politics-specific loss reminder should mobilize participation

The literature on collective action shows that perceptions of injustice can mobilize engagement by eliciting group-based anger, strengthening politicized social identity, and bolstering collective efficacy, all of which have been associated with increased political behaviors such as voting, organizing, and engaging in political discussions [22,29,30]. For example, in marginalized populations, perceptions of discrimination often lead to increased political participation through these pathways [19,21,31]. Among American Indian adults specifically, perceptions of race-related discrimination have been associated with increased political participation in part through internal and collective political efficacy [23]. Because the USPLRS contextualizes historical losses within a contemporary political lens (“U.S. politics reminds me of…”), it should activate these action-relevant appraisals compared with the HLS, which measures the frequency of thoughts related to loss in general and is often studied in relation to distress and health [1]. Thus, we expect the USPLRS to exhibit incremental validity for political participation beyond the HLS.

### U.S. politics as a unique loss reminder

American Indians have endured a history riddled with severely discriminatory practices by the U.S. government, and this discrimination continues today [32]. Thus, U.S. politics may act as a unique and powerful reminder of the deep-rooted historical losses suffered by American Indians. In their introduction of the HLS, Whitbeck et al. [1] found that nearly one-third of respondents reported at least sometimes avoiding people and places that reminded them of historical losses. However, the degree to which U.S. politics specifically acts as a reminder of historical losses has not been examined. Consequently, it is important to measure the extent to which U.S. politics prompts thoughts about HL and to evaluate whether this association predicts political participation. Building on this rationale, the current study introduces the U.S. Politics as a Loss Reminder Scale (USPLRS) to operationalize these politics-specific reminders.

### U.S. Politics as a Loss Reminder Scale (USPLRS)

To measure this politics-specific reminder, the current study introduces the U.S. Politics as a Loss Reminder Scale (USPLRS), which measures the degree to which contemporary U.S. politics reminds individuals of historical losses, in contrast to the HLS which focuses on the general frequency of historical-loss thoughts.

The Historical Loss Scale (HLS) is a widely used tool for measuring historical loss among American Indians. The HLS was originally explained by a single latent factor that accounted for 58% of the variance. However, a later longitudinal study revealed a 3-factor structure, with subsets of items tapping into general loss, loss of people, and cultural mistreatment [4]. Items conceptualized as general loss were those related to the loss of land, language, spiritual ways, and culture. The items associated with the loss of people contained one item regarding the loss of life because of alcoholism, and one item regarding loss due to early deaths. Items associated with cultural mistreatment were the loss of family ties, families, self-respect, and trust in whites [4].

Research utilizing the HLS has mainly focused on the relationships between the frequency of thoughts about historical loss, and negative health outcomes [7,8,33]. By placing the items in the context of U.S. politics, the USPLRS may help uncover unique associations between historical loss and political engagement that may be more behavior-proximal and more salient for individuals who are politically interested or knowledgeable [34]. Rather than asking the frequency with which American Indians think about losses, the USPLRS specifically asks the degree to which U.S. politics reminds them of those losses, providing a context-specific indicator of the cognitive link between current U.S. politics and historical loss. Demonstrating the factor structure, reliability and incremental validity (i.e., unique prediction beyond the HLS) of the USPLRS would support the adaptability of the HLS to other meaningful contexts (e.g., exposure to mass or social media, socioeconomic context, local/community gatherings, or participation in traditional ceremonies.

### Conceptual distinction and nesting of HLS and USPLRS

While the HLS measures the frequency of thoughts about historical losses (e.g., loss of land, language, culture, etc.) [Whitebeck et al., 2004], the USPLRS assess context-specific associations, specifically the degree to which contemporary U.S. politics *reminds* respondents of those losses. Thus, the USPLRS is not a subset of the original HLS, but a complementary construct. The HLS capture base-rate salience of loss cognitions, while the USPLRS captures politics-cue reactivity to the same content domain. This distinction is important for political behavior because politically-framed appraisals (e.g., injustice, politicized social identity, collective efficacy) are theoretically the most relevant to political behavior [19,21–23,29–31]. Therefore, the current study tests whether the USPLRS demonstrates incremental validity for political participation over and above the HLS. Finally, some HLS contents do not need to be cued by U.S. politics (e.g., losses from the effects of alcoholism, early deaths, loss of respect by children for elders), whereas others more directly implicate government policy (e.g., relocation, broken treaties [1,4].

Linked fate, or the belief that one’s life chances and outcomes are tied to the fate of the group, is a well-established predictor or political participation in minority groups [35–37]. In American Indian samples, the findings are relatively mixed. Some research suggests the importance of group and sovereignty dynamics associated with linked fate, whereas other studies show weaker or null effects of group consciousness and linked fate on participation relative to other groups (Huyser et al., 2016; [38]). The USPLRS is conceptually related with distinct from linked fate. Rather than assessing generalized group-based fate, the USPLRS measures whether current U.S. politics cues historical losses. We suggest this politics-specific appraisal is especially relevant for political behavioral outcomes.

### Current study

Previous research has established the reliability and validity of using the HLS to measure frequency of thoughts about historical loss in American Indians [1]. However, because American politics may be a unique reminder of historical loss, the current study introduces an adaptation of the HLS, called the USPLRS, which contextualizes historical losses within U.S. politics, and measures the degree to which U.S. politics can act as a reminder of these losses. Specifically, rather than focusing on the quantitative aspects of historical loss, as is measured by the original HLS, the USPLRS emphasizes the qualitative aspects of historical loss within the context of U.S. politics. In doing so, the current study aims to provide a more nuanced understanding of how a certain context (in this case U.S. politics) can directly trigger thoughts related to historical loss. Thus, the primary purpose of this study was to assess the factor structure of the USPLRS and explore its relationship with political engagement among American Indians. First, an exploratory factor analysis (EFA) was conducted on responses to the USPLRS to examine its factor structure. Next, a confirmatory factor analysis (CFA) was conducted to examine the fit of the factor structure.

To assess the predictive validity of the scale, we then examined the relationship between scores on the USPLRS and levels of political engagement. To determine whether the USPLRS is an independent predictor of political engagement, above and beyond the HLS, we included scores on the HLS when investigating whether the USPLRS predicts political engagement. Based on previous research on the relationship between perceived injustices and discrimination and political participation in other groups [19–21], we hypothesized that the USPLRS, which assesses the extent to which U.S. politics acts as a reminder of historical losses, would be positively related to political engagement among American Indian adults.

## Methods

### Participants

The current study was approved by the X Institutional Review Board. All participants provided informed consent prior to participation in the study. Participants for this study were recruited through Qualtrics. Qualtrics Panels draw participants from managed research panels for groups that are more difficult to reach, which are developed through targeted recruiting. To be eligible for the study, participants had to currently live within the U.S., identify as American Indian, and be over the age of 18. Based on these eligibility criteria, the current study utilizes a sample of 877 self-identified American Indians over the age of 18 recruited through a Qualtrics panel. Self-identification as American Indian was determined by one self-report item: “what is your race/ethnicity? (Select all that apply).” Participants who selected “Native American/American Indian” were included in the current study,

All data were collected by Qualtrics in November, 2020, and were screened for quality before being sent to investigators. This data screening included removing participants who completed the survey in less than half of the median time of survey completion. After data screening, all deidentified data were sent to the investigators in an excel file, and subsequently transferred to SPSS (IBM, version 28) for analyses.

### Measures

#### Demographics.

Participants were asked to self-report basic demographic information including age, gender, and income. Participants were between 18–84 years old (*M* = 37.68 years; *SD* = 15.24 years). 597 participants (70.1%) identified as female. Average family income was approximately $42,000. Based on zip code data, the current sample contained participants from all regions of the U.S. (Northeast, Midwest, South, and West), indicating broad geographic coverage.

### Historical Loss Scale (HLS)

The HLS is a 12-item self-report measure used to assess the frequency with which American Indians think about historical losses. Participants respond on a 6-point Likert scale ranging from 1 = several times a day to 6 = never, and are reverse-scored, such that higher scores indicate more frequent thoughts about historical loss. Participants report their frequency of thoughts regarding specific losses including the loss of land, traditional values, trust, culture, respect, and the loss of life (e.g., “Please rate how often you think about each type of loss: the loss of our land”). Responses to each item are summed to obtain a total score reflecting the overall frequency with which individuals think about these losses (α = .95).

### U.S. Politics as a Loss Reminder Scale (USPLRS)

This questionnaire was adapted from the original Historical Loss Scale (HLS) developed by Whitbeck and colleagues [1]. Specifically, the USPLRS utilizes the same 12 items related to historical losses (e.g., loss of land, loss of culture, etc.) as the original HLS. However, the HLS asks participants to report the extent to which they think about these losses (e.g., Please indicate how often you think about each type of historical loss. – The loss of our land). Responses to the HLS are 1 = several times a day to 6 = never and are reverse-scored, such that higher scores indicate more frequent thoughts about historical loss. In the USPLRS, items are framed as: “The next questions are about U.S. politics and historical loss. Rate your agreement with the following statements. – U.S. politics reminds me of the loss of our land.” Responses range from 1 = strongly disagree to 5 = strongly agree. By framing the historical loss items from the HLS within the context of U.S. politics, the USPLRS can assess the extent to which U.S. politics act as a reminder of historical losses. Further, by asking participants their level of agreement with each statement, we can assess more qualitative aspects of the historical loss items, as opposed to the quantitative nature of the original HLS which is based on the frequency of thoughts related to historical losses. Responses to all items are summed to create a total score, with higher scores reflecting a stronger link between U.S. politics and historical losses (α = .95).

### Political and community involvement

This scale has been adapted from previous research, which includes items regarding engagement in all three types of civic engagement activities within the past month [39]. Of interest to the current study, are items related to political engagement, and thus, those are the only items included in analyses.

*Standard Political Participation* Participants self-reported their engagement in each political activity within the past month with responses ranging from 1 = Never to 5 = Multiple times a day. Higher scores indicate more involvement in the activity of interest. Six items were included which measured frequency of participation in activities such as expressing political opinions, reading political news, watching political news, discussing politics with friends and family, and participating in a political party, club, or organization (e.g., “Please rate how often you have participated in each activity in the past month: Participated in a political party, club, or organization”).

## Results

### Factor analyses

To examine the factor structure of the USPLRS and select items for a final scale, we conducted an exploratory (EFA) and confirmatory factor analysis (CFA). We used a random number generator to divide our total sample into a test sample (*N* = 439) and a confirmatory sample (*N* = 438). This procedure of randomly dividing a total sample into EFA and CFA subsamples is a commonly used approach which ensures the structure identified in the EFA is tested and confirmed in an independent sample through CFA, providing enhanced robustness and generalizability of the factor analysis (e.g., [40–43]). The samples did not significantly differ in terms of demographics.

#### Exploratory Factor Analysis (EFA).

To examine the factor structure and to select items for the final scale, we first conducted an exploratory factor analysis. The Kaiser-Meyer-Olkin (KMO) measure of sampling adequacy was .96, indicating suitability for factor analysis [44]. Prior research has shown the items on the HLS to be correlated with one another, we thus opted for a Promax rotation to allow potential factors to correlate [1,4]. A parallel analysis (PA) with Promax rotation was conducted to determine the number of factors to retain. First, we assessed the one-factor structure. The model was a good fit and explained 64% of the variance, with factor loadings ranging from .76 to .83. Consistent with previous research showing the HLS can be explained by a 3-factor solution with factors relating to cultural loss, the loss of people, and cultural mistreatment [4], PA suggested a 3-factor solution. One item (Item 3: “U.S. politics reminds me of the loss of our traditional spiritual ways”) had cross loadings on multiple factors, and thus was excluded in subsequent analyses. The 3-factor solution explained 67% of the variance. Factor 1 had an eigenvalue of 3.36 accounting for 31% of the variance. Factor 2 had an eigenvalue of 2.21 and accounted for 19% of the variance. Factor 3 had an eigenvalue of 1.86 accounting for 17% of the variance. Factor loadings ranged from.40 to.96, indicating good item-to-factor fit, and are depicted in Table 1.

**Table 1:** Exploratory Factor Analysis (EFA).

USPLRS item	Factor loading		
	1	2	3
Factor 1: Government Mistreatment			
1. U.S. politics remind me of the loss of our land.	**.94**	−.04	−.08
2. U.S. politics remind me of the loss of our language	**.40**	.27	.14
5. U.S. politics remind me of how we lost families from the reservation to government relocation.	**.69**	.16	.01
6. U.S. politics remind me of how we lost self-respect from poor treatment by government officials	**.60**	.11	.17
7. U.S. politics remind me of how we lost trust in whites because of broken treaties. 8. U.S. politics remind me of the loss of our culture.	**.68** **.64.**	.10 −.04	.03 .23
Factor 2: Interpersonal Loss			
4. U.S. politics remind me of how we lost family ties because of boarding schools.	.10	**.45**	.24
9. U.S. politics remind me of the losses from the effects of alcoholism on our people.	−.03	**.86**	.04
11. U.S. politics remind me of the loss of our people through early death.	.12	**.74**	−.03
Factor 3: Respect			
10. U.S. politics remind me of the loss of respect by our children and grandchildren for elders	.01	.08	**.76**
12. US politics remind me of the loss of respect by our children for traditional ways.	.05	.00	**.82**

**Fig 1:**
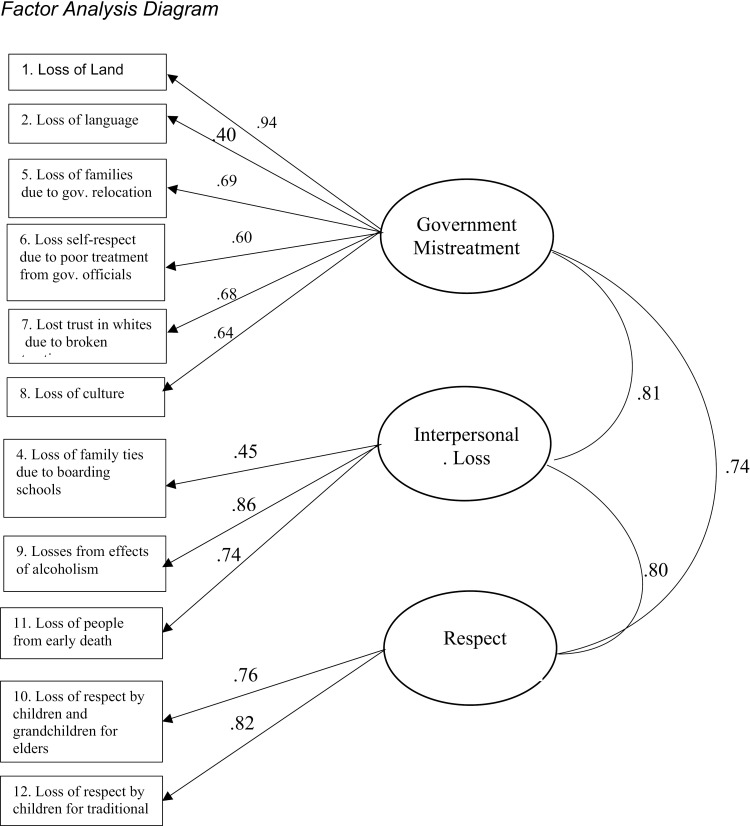
Factor analysis diagram.

The factors were named based on the items with the highest loading on each factor. Factor 1, labeled “Government Mistreatment” included general losses related to governmental policies and mistreatment (e.g., loss of land, loss of language, loss of culture, loss due to government relocation, loss of self-respect due to poor treatment from government, loss of trust due to broken treaties). Factor 2, labeled “Interpersonal Loss” included 3 items pertaining to interpersonal losses (e.g., loss of family ties due to boarding schools, losses from effects of alcoholism, loss of people through early death). Factor 3, labeled “Respect” included 2 items related to loss of respect (e.g., loss of respect by children for elders, loss of respect by children for traditional ways). Factor names, and factor loadings for the retained items are presented in Table 1. A diagram of the factor analysis including factor loadings and correlations between factors is depicted in Figure 1.

#### Confirmatory Factor Analysis (CFA).

A CFA was conducted on the confirmatory sample to test the fit of the 3-factor solution identified in the EFA. Results indicate a 3-factor solution with item loadings ranging from .76 to .87. Model fit indices are summarized in Table 2. Absolute fit indices included the chi-square statistic (χ² = 44.57, df = 25, *p* <.0094), RMSEA = .062, and SRMR = .024. Due to large samples often resulting in significant chi-square values [45], we used the relative chi-square approach (χ 2/df) suggested by Wheaton et al. [46]. The relative chi-square was 2.78, which falls in the acceptable cutoff range of 2.0 to 5.0 [47]. Incremental fit indices suggest the model fits the data well (CFI = .981 and TLI = .975). The minimum correlation of possible factor scores is also high, indicating that the factors are well-defined and distinct from each other. Finally, each factor demonstrated high internal validity. The internal consistency and reliability was assessed using Cronbach’s alpha coefficients which were .94, .84, and .84 for each respective factor. These values suggest that items within each factor are tapping into the same construct.

**Table 2:** Model Fit Indices for Confirmatory Factor Analysis (CFA).

Fit Index	Value	Cutoff for good fit	Good/Poor fit
Chi-square (df = 41)	114.09	Nonsignificant p-value	Poor (p < .001)
Relative chi-square (χ 2/df)	2.78	2.0 <(χ 2/df) < 5.0	Good
Root Mean Square Error of Approximation (RMSEA)	.062	<.10	Good
Standardized Root Mean Square Residual (SRMR)	.024	<.05	Good
Comparative Fit Index (CFI)	.981	>.90	Good
Tucker-Lewis Index (TLI)	.974	>.90	Good

### Descriptive statistics

Descriptive statistics for the variables of interest are presented in Table 3.

**Table 3:** Descriptive statistics for variables of interest.

Variable	N	Mean (%)	Std. Deviation	Range
Age	877	37.82	15.17	18-84
Gender	852	70.1%(Female)	.473	1-2
Income	850	$41,929.41	$35,694.95	$5,000-$150,000
Political Participation	877	15.73	5.47	6-30
HLS	877	44.40	20.28	12-72
Government Mistreatment	877	3.44	6.05	6-30
Interpersonal Loss	877	3.21	3.13	3-15
Respect	877	3.30	2.19	2-10
USPLRS Total	877	3.35	10.59	11-55

### Bivariate correlations

Bivariate correlations of the variables of interest are presented in Table 4. In line with previous research, income was associated with political participation (*r* = .220, *p* < .001) [25**]**. However, neither age, nor gender were associated with political participation. Scores on the HLS were positively associated with political participation (*r* = .089, *p* < .001), suggesting American Indian adults in this sample who think frequently about historical losses participate more in politics.

**Table 4:** Bivariate correlations.

Variables	1.	2.	3.	4.	5.	6.	7.	8.
**1. Age**								
**2. Gender**	−.019							
**3. Income**	.106**	.106**						
**4. Pol. Participation**	−.068	−.049	.196**					
**5. HLS**	−.500	.096**	−.031	.136**				
**6. Gov. Mistreatment**	.061	.129**	.026	.197**	.394**			
**7. Interpersonal Loss**	.035	.091**	.019	.160**	.397**	.804**		
**8. Respect**	.108**	.088^*^	.032	.161**	.366**	.772**	.751**	
**9. USPLRS Total**	.067^*^	.118**	.027	.193**	.418**	.968**	.909**	.869**

* p <.05, **p <.001.

Beyond establishing the reliability and validity of the USPLRS, we were also interested in examining the relationship between scores on the USPLRS and political engagement among American Indian adults. To examine this relationship, we performed correlation analyses of each individual factor in relation to political engagement, as well global scores on the scale and political engagement. Government Mistreatment was positively associated with political engagement (*r* = .197, *p* < .001), suggesting that individuals who reported U.S. politics reminds them of general losses related to government mistreatment were more likely to engage in U.S. politics. Interpersonal Loss was also positively associated with political engagement (*r* = .160, *p* < .001), suggesting that individuals who reported US politics reminds them of interpersonal losses were more likely to engage in US. politics. Respect was positively associated to political engagement (*r* = .161, *p* < .001) suggesting that individuals who reported U.S. politics reminds them of losses related to the loss of respect were more likely to engage in U.S. politics. Finally, total scores on the scale were positively associated with political engagement (*r* = .193, *p* < .001) suggesting that individuals who reported that U.S. politics reminds them of each type of loss were more likely to engage in U.S. politics.

### Regression analysis

We conducted a hierarchical regression analysis to examine the unique contributions of the USPLRS and HLS in predicting political participation, while controlling for age, gender, and income. The results of this analysis are presented in Table 5.

**Table 5:** Hierarchical regression analyses for political participation.

Dependent Variable: Political Participation				
Independent Variables	β	F	*R* ^2^	Δ R²
Model 1: Demographic Variables		14.801**	.047	
Age	−.033**			
Gender	−.333			
Income	.355**			
Model 2: Addition of HLS		15.888**	.066	.019
Age	**−.030** ^*^			
Gender	−.489			
Income	.359**			
HLS	.038**			
Model 3: Addition of USPLRS		18.404**	.093	.027
Age	−.036**			
Gender	−.673			
Income	.347**			
HLS	.018			
USPLRS	.097**			

* p <.05, ** p <.001.

#### Incremental validity.

Including the USPLRS in the final step yielded a significant increase in explained variance in political participation (Δ R² = .027), indicating incremental validity over the HLS. In the full model, the USPLRS was a significant predictor of political participation (β =.097, *p* < .001), whereas the HLS was not (β =.018, *p* = .072).

#### Model steps.

Step 1 (covariates: age, gender, income) was significant *F* (3, 846) = 14.801, *p* <.001, *R*^2^ = .047). Step 2 (adding HLS) was also significant, *F* (4, 845) = 15.888, *p* <.001, *R*^2^ = .066 (Δ*R*^2^ = .019). Step 3 (adding USPLRS) was significant, *F* (5, 844) = 18.404, *p* <.001, *R*^2^ = .093 (Δ*R*^2^ =.027). These results indicate that the USPLRS provides unique explanatory power for political participation beyond demographics and the HLS.

### General discussion

The current study introduced and validated a novel adaptation of the original HLS called the USPLRS which measures the extent to which U.S. politics acts as a reminder of historical losses.

An EFA was conducted to examine the factor structure of USPLRS. This analysis first revealed a one factor structure that was a good fit, explaining 64% of the variance. In line with previous research which assessed a 3-factor structure of the HLS, we also examined a 3-factor structure of the USPLRS [4]. The 3-factor structure was strong, explaining 67% of the variance. Item 3 was removed due to high cross-loadings across all factors. The 3 factors were named based on the items that had high loadings on each factor: Government Mistreatment, Interpersonal Loss, and Respect. We elected to retain this 3-factor structure under the notion that losses related to government mistreatment, interpersonal loss, and respect may have differential predictive validity in relation to political participation and other potential outcomes. The CFA supported the 3-factor structure with sufficient absolute (RMSEA = .062; SRMR = .024) and incremental fit indices (TLI = .974; CFI = .981). The USPLRS exhibited strong internal consistency, with Cronbach’s alpha ranging from .84 to .92 for each factor. The results of this study provide evidence for the reliability and validity of the USPLRS in the extent to which U.S. politics act as a historical loss reminder. The 3-factor structure also suggests that losses related to government mistreatment, interpersonal losses, and respect are distinct from one another.

The predictive validity of the scale was examined regarding its relationship to political engagement. Scores on each factor (Government Mistreatment, Interpersonal Loss, and Respect) were each positively associated with political participation. Similarly, global scores on the USPLRS were positively associated with political participation. These results suggest that the individual factors, as well as the total USPLRS, are not only effective measures of the degree to which U.S. politics as a reminder of historical losses, but are also predictive of political participation.

The full model accounted for 9.3% of the variance in political participation, and the addition of the USPLRS increased *R*^2^ by .027. As a single, theoretically grounded construct predicting a broad range of non-electoral participation above and beyond the HLS, this incremental validity obtained from the addition of the USPLRS is modest but meaningful. The primary aim of the current study was to validate the USPLRS and test incremental validity over the HLS, rather than to build a maximally predictive model that includes all standard covariates (e.g., political interest/knowledge, internal and collective efficacy, linked fate, etc.).

The current findings help shed light on why the USPLRS predicted political participation. Research utilizing the HLS has consistently shown that greater thoughts related to historical loss are associated with increased symptoms (e.g., anxiety, depression, PTSD) that often reduce engagement [5,7,28]. On the other hand, the USPLRS frames those losses in a contemporary political lens that may activate action-relevant appraisals such as perceived injustice, politicized social identity, and collective efficacy, which have been shown to mobilize participation [22,29,30], including among American Indian adults where perceptions of race-related discrimination has been associated with increase political participation through political efficacy [23]. Since we did not measure these potential intervening processes in the current study, we suggest them as testable mechanisms for future research.

To our knowledge, the current study was also the first to examine the relationship between the HLS and political participation. American Indian adults in this sample who thought more frequently about historical losses reported higher levels of political participation (*r* =.136, *p* <.001). As shown in Table 4, scores on the HLS were positively associated with USPLRS scores on Government Mistreatment (*r* =.394, *p* <.001), Interpersonal Loss (*r* =.397, *p* <.001), Respect (*r* =.366, *p* <.001), and total scores on the USPLRS (*r* =.418, *p* <.001). The positive association between the HLS and USPLRS (*r* =.418, *p* <.001) is not surprising given the overlap of the context of items on both scales, yet the magnitude of correlations were not high enough to suggest the USPLRS is redundant with the HLS. Further, data were collected in November 2020 during the U.S. national election cycle. Thus, if U.S. politics were salient during this time, and reminded individuals of historical losses, it is likely they also had more frequent thoughts about historical losses. Alternatively, if individuals were having frequent thoughts about historical losses during this time, it may also be likely that U.S. politics could trigger a reminder of these losses. Notably, the USPLRS exhibited statistically significant incremental validity beyond the original HLS in relation to political participation. Thus, the USPLRS may provide a more salient and relevant measure of historical loss in the context of U.S. politics than the original HLS and may be used as a more sensitive tool in predicting political participation among American Indian adults.

Overall, these patterns support the view that the HLS and the USPLRS tap into related but distinct aspects of historical loss. The HLS reflects the general frequency (base-rate salience) of thoughts related to historical loss, whereas the USPLRS captures politics-specific reminders (cue reactivity) of those losses. Conceptually, many HLS contents can be activated in non-political contexts (e.g., family interactions, alcohol-related harms), while the USPLRS targets a political context that theory and empirical evidence identifies as especially proximal to civic action and political participation [22]; Postmes & Spears, 2008; [19,21,23,29–31]. Importantly, the current study frames the USPLRS as complementary and more relevant for political outcomes, rather than globally “better” than the HLS. For example, the HLS may be optimal for domains such as health-related distress, while the USPLRS may be optimal for domains related to civic and political behavior.

Consistent with this idea, studies across marginalized groups show that perceived discrimination and injustice can mobilize political action through increasing saliency of politicized identity and bolstering efficacy [19,21,31,48,49]. Among American Indian adults specifically, research has shown that race-related perceived discrimination predicts political participation in part through internal and collective political efficacy [23]. At the same time, effects can be null or even negative when efficacy and resources are low, or when discrimination is individualized rather than collective [50,51]. Together, these patterns situate the USPLRS as a politics-specifc reminder measure that is especially relevant for civic and political behavioral outcomes.

### Implications

Overall, the results of the current study provide evidence for the reliability and validity of the USPLRS. The USPLRS provides a novel perspective on the impact of historical loss and trauma by investigating the link between U.S. politics and historical losses, and investigating whether the strength of this link predicts levels of political participation in American Indians. The USPLRS scale could be used to predit other relevant political outcomes including voting behavior. In line with this, other forms of civic engagement that have political implications, such as social movement activities (e.g., attending a protest) may be affected by the degree to which U.S. politics acts as a reminder of historical losses. Given the positive associations between scores on the USPLRS and political engagement, the scale could be used in future research to identify American Indians that are more or less likely to engage in politics. Given nomothetic-idiographic disjunction, the findings of the current study, which utilized a large-scale survey, are difficult to apply to individual cases [52]. However, the current findings can be utilized to identify general patterns in terms of the relationship between U.S. politics acting as a reminder of historical losses and political participation among American Indian adults. From there, in-depth interviews may be useful to explore these relationships among individuals in detail. The combination of nomothetic and idiographic approaches can thus be utilized to provide a more enriched and nuanced understanding of these phenomena.

Interventions could target individuals who are likely to engage in politics and provide them with ample opportunities for participation. Alternatively, individuals that are less likely to participate can be targeted to increase levels of motivation to participate.

### Limitations

There are several limitations of the current study. First, the data collected was cross-sectional, and thus, causation cannot be established. Future research would benefit from the use of longitudinal designs to examine the relationships between historical loss, historical loss reminders, and political engagement. Additionally, the data was collected in November 2020 during the national election cycle. It is likely that during an election cycle, individuals were simply more engaged in politics relative to other times, which could have influenced the results. Specifically, during a time in which individuals are at a heightened level of political engagement, questions asking about U.S. politics may have been more salient, simply due to the heightened salience of politics during a national election cycle. Despite this limitation, participants with greater scores on the USPLRS still reported greater levels of political participation in the current study. Additionally, overall explanatory power was modest (*R*^2^ =.093), and correlations with participation were relatively small (see Table 4). The current study also did not include several standard predictors used in research on political participation (e.g., political interest/knowledge, internal and collective efficacy, linked fate) [10,13,25,35–38]. While including these variables may have increased overall model fit, their exclusion in the current study means the present analyses provide a conservative test of validity for the USPLRS relative to the HLS.

Further, notions that U.S. politics acting as a loss reminder can be conceptualized as a perception of injustice/discrimination are speculative as this relationship was not directly measured in the current study. This speculation may influence the interpretation of the current findings in terms of perceptions of injustice/discrimination acting as a motivator for political participation. Specifically, the relationship between scores on the USPLRS and political participation may work through some other mechanism other than perceptions of injustice/discrimination. However, given that historical losses are inherently discriminatory [4], this conceptualization may be justified. Nonetheless, future research would benefit from directly examining the extent to which U.S. politics as a loss reminder are perceived as an injustice/discrimination. Finally, forms of political engagement were not specified in terms of local and tribal politics and national politics. Thus, the results may have partially been due to American Indians participating in local and tribal politics, rather than U.S. politics.

Despite these limitations, the results suggest that the USPLRS is a reliable and valid instrument for measuring the extent to which U.S. politics act as reminder of historical losses. Interestingly, the results suggest that loss reminders in the context of U.S. politics are associated with greater political participation among American Indian adults.

### Future directions

These findings suggest that a stronger connection between U.S. politics and historical injustices relate to greater political engagement among American Indians. Future research would largely benefit from longitudinal studies to reveal causal and temporal relationships between historical losses, the extent to which U.S. politics act as a reminder of these losses, and political participation.

We also recognize potential boundary conditions under which reminders could reduce participation. For example, when political efficacy is low, resources are limited, or reminders lead to avoidance, reminders of HLS, including those in the context of U.S. politics could reduce participation. Further, research has demonstrated contexts in which perceptions of discrimination do not lead to engagement, but rather hinder it (e.g., [31,50]. Future longitudinal research should test mediation and moderation via mechanisms such as group-based anger, politicized social identity, collective efficacy, linked fate and examine political knowledge/interest, and resource factors associated with participation [9,10,13,25,35–38,48,53,54]. Further, future research would benefit from incorporating these standard predictors to provide a baseline estimate of overall explanatory power (e.g., total/adjusted *R*^2^), as well as to evalute whether the USPLRS continues to explain unique variance beyond these factors (Δ*R*²).

Future research would also benefit from examining if effects differ for national versus tribal/local political participation and for individual versus group-level discrimination. It would also be beneficial to examine the relationship between historical losses, voting behavior, and other forms of civic engagement, such as community service, and social movement activities. Further, a deeper analysis of these relationships among individuals, through the use in-depth interviews and other methodologies, can provide a more nuanced understanding of how these relationships can be harnessed in the development of interventions to bolster American Indian political participation. Finally, future research would benefit from directly examining the extent to which historical losses are perceived as experiences of discrimination and how these experiences influence a sense of racial and ethnic identity.

## Conclusion

The current study provides evidence for the reliability and validity of an adaptation of the original Historical Loss Scale (HLS) titled the U.S. Politics as a Loss Reminder Scale (USPLRS). An analysis of the factor structure of the USPLRS revealed three distinct factors associated with losses related to government mistreatment, interpersonal loss, and respect. In a sample of American Indian adults, thoughts about historical losses and the extent to which U.S. politics act as a reminder of these losses, was positively associated with political participation. Importantly, the USPLRS exhibited incremental validity for political participation beyond the original HLS.

To our knowledge, the current study is the first to examine the link between U.S. politics and thoughts about historical losses, and the first to test whether the strength of this relationship predicts political participation in American Indian adults [10]. The results have potential implications for the development of interventions and campaigns to promote political participation among American Indian populations, a population with traditionally lower levels of political participation.

## Supporting information

S1 File USPLRS ZIP removed. (XLSX)
